# Seed-Specific Silencing of Abundantly Expressed Soybean Bowman–Birk Protease Inhibitor Genes by RNAi Lowers Trypsin and Chymotrypsin Inhibitor Activities and Enhances Protein Digestibility

**DOI:** 10.3390/ijms26146943

**Published:** 2025-07-19

**Authors:** Wonseok Kim, Sunhyung Kim, Hari B. Krishnan

**Affiliations:** 1 Division of Plant Science and Technology, University of Missouri, Columbia, MO 65211, USA; wonseokk@missouri.edu (W.K.); jeongs@missouri.edu (S.K.); 2 Plant Genetics Research Unit, US Department of Agriculture-Agricultural Research Service, Columbia, MO 65211, USA

**Keywords:** soybean, *Glycine max* (L. (Merr)), antinutritional factors, Bowman–Birk inhibitor, RNA interference, confocal fluorescence immunolabeling, protein digestibility

## Abstract

Soybean meal (SBM) is extensively used as a predominant protein source in animal feed. However, raw soybean cannot be directly utilized in animal feed, due to the presence of the Kunitz trypsin inhibitor (KTi) and the Bowman–Birk protease inhibitor (BBi). These antinutritional factors inhibit the digestive enzymes in animals, trypsin and chymotrypsin, resulting in poor animal performance. To inactivate the activity of protease inhibitors, SBM is subjected to heat processing, a procedure that can negatively impact the soybean protein quality. Thus, it would be beneficial to develop soybean varieties with little or no trypsin inhibitors. In this study, we report on the creation of experimental soybean lines with significantly reduced levels of Bowman–Birk protease inhibitors. RNA interference (RNAi) technology was employed to generate several transgenic soybean lines. Some of these BBi knockdown soybean lines showed significantly lower amounts of both trypsin and chymotrypsin inhibitor activities. Western blot analysis revealed the complete absence of BBi in selected RNAi-derived lines. RNA sequencing (RNAseq) analysis demonstrated a drastic reduction in the seed-specific expression of *BBi* genes in the transgenic soybean lines during seed development. Confocal fluorescence immunolabeling studies showed that the accumulation of BBi was drastically diminished in BBi knockdown lines compared to wild-type soybeans. The absence of BBi in the transgenic soybean did not alter the overall protein, oil, and sulfur amino acid content of the seeds compared to wild-type soybeans. The seed protein from the BBi knockdown lines were more rapidly hydrolyzed by trypsin and chymotrypsin compared to the wild type, indicating that the absence of BBi enhances protein digestibility. Our study suggests that these BBi knockdown lines could be a valuable resource in order for plant breeders to incorporate this trait into commercial soybean cultivars, potentially enabling the use of raw soybeans in animal feed.

## 1. Introduction

Soybean is one of the most important crops that accumulates high amounts of protein and oil in its seeds [1]. Its protein and oil content are about 40 and 20%, respectively. Most of the soybeans produced in the US are primarily used in animal feed. Soybeans are processed to first extract oil and residual protein meal is subjected to additional processing to yield defatted soybean meal. Soybean meal (SBM), with its complete amino acid profile, is an excellent protein source for livestock [2]. Importantly, soybeans are very productive and potentially more environmentally sustainable, due to their ability to fix nitrogen in association with soil-dwelling rhizobia. Despite these advantages, SBM cannot be directly used for animal feed mixtures, due to the presence of various antinutritional compounds, which reduce animal weight gain [3,4,5].

Among the antinutritional compounds identified in soybeans, proteinase inhibitors pose a challenge due to their interference with the digestive enzymes in animals, specifically trypsin and chymotrypsin [6,7,8]. The two major proteinase inhibitors in soybean seeds are the Kunitz trypsin inhibitor (KTi) and the Bowman–Birk inhibitor (BBi). KTi specifically inactivates trypsin, whereas BBi inhibits both trypsin and chymotrypsin [6,7,8]. Soybeans contain three KTi isoforms, KTi1, KTi2, and KTi3, with KTi3 being the most abundant [9]. In contrast, five BBi isoforms, A, B, C-II, D-II, and E-1 have been isolated from soybean seeds [8,10]. BBi-A and BBi-B exhibit similar inhibitory specificity and amino acid composition [8]. In silico and quantitative RT-PCR analyses have identified 11 potential BBi genes in the soybean genome [11], as detailed in Supplementary Table S1. Among these, three genes, *BBi*-A, *BBi*-CII, and *BBi*-DII, are specifically expressed in seeds, with *BBi*-DII transcripts being the most abundant, followed by *BBi*-A and *BBi*-CII [11]. Both KTi and BBi proteins can be irreversibly inactivated by heat, although BBi proteins exhibit greater resistance to heat inactivation compared to KTi proteins [3]. Consequently, soybean seed meal undergoes routine heat processing, which can reduce protein quality and digestibility due to the Maillard reaction [12].

Germplasm screening has identified soybean accessions with reduced or absent Bowman–Birk (BBi) or Kunitz trypsin inhibitor (KTi) proteins. One such accession, PI 157740, exhibits significantly reduced (~40%) trypsin inhibitor activity [13]. Molecular cloning and sequencing of this mutant revealed a frameshift mutation in the *KTI*3 coding region [14]. This accession has been extensively backcrossed and subjected to feeding trials, which demonstrated that raw, extruded protein meal lacking KTI3 enhances animal weight gain compared to raw soybeans [5,15,16]. In addition, another PI line (PI 68679) was identified with a frameshift mutation in the *KTI1* coding region. Through conventional hybridization, this line was crossed with the *KTI*3-deficient line, generating an experimental soybean line deficient in both KTI1 and KTI3 proteins [17]. This double mutant exhibited a pronounced reduction in trypsin inhibitor activity. Nevertheless, all mutant lines retained substantial protease inhibitor activity, due to the continued presence of Bowman–Birk inhibitors.

Although progress has been made in reducing the trypsin inhibitor content of soybean seeds, no soybean cultivars have been released that are entirely devoid of trypsin inhibitor activity. This is primarily due to the presence of the Bowman–Birk protease inhibitor (BBi), which inhibits both chymotrypsin and trypsin. To date, no *Glycine max* (*G. max*) germplasm has been identified with a complete or consistent reduction in seed-expressed *BBi* genes. However, Bowman–Birk protease inhibitor mutants have been found in several perennial *Glycine* species [18]. A *Glycine microphylla* (*G. microphylla*) accession with a single loss-of-function frameshift mutation affecting one of the *BBi* genes has been identified [19]. Due to substantial hybridization barriers between *G. microphylla* and *G. max*, the BBi-null trait has not been successfully transferred to *G. max*.

Efforts to reduce BBi protein levels in soybean seeds have been pursued through genetic engineering. Nelson and colleagues overexpressed a mutated, inactive BBi transgene, which successfully outcompeted endogenous BBi transcripts, leading to a significant reduction in BBi protein levels [20]. More recently, CRISPR/Cas9 gene editing technology has been employed to introduce mutations in *BBi* genes, drastically lowering the protease inhibitor content in soybean seeds [21]. This study demonstrated that mutations in two highly expressed, seed-specific *BBi* genes resulted in substantial reductions in both trypsin and chymotrypsin inhibitor activities. However, even in these CRISPR/Cas9-modified lines, trypsin and chymotrypsin inhibitor activities were not completely eliminated [21].

Since eliminating the BBi would enhance the nutritive value of soybeans for both animal feed and human consumption, developing soybean lines with minimal or no Bowman–Birk protease inhibitors remains a desirable goal. As an alternative to CRISPR/Cas9 gene editing, we have successfully employed RNA interference (RNAi) technology to downregulate the expression of the most abundant *BBi* genes in soybean seeds. RNAi-generated BBi mutant plants exhibited a drastic reduction in both trypsin and chymotrypsin inhibitor activities. Furthermore, the elimination of the BBi improved the digestibility of soybean seed proteins.

## 2. Results

### 2.1. Creation of BBi Knockdown Transgenic Soybean Plants

**Figure 1:**
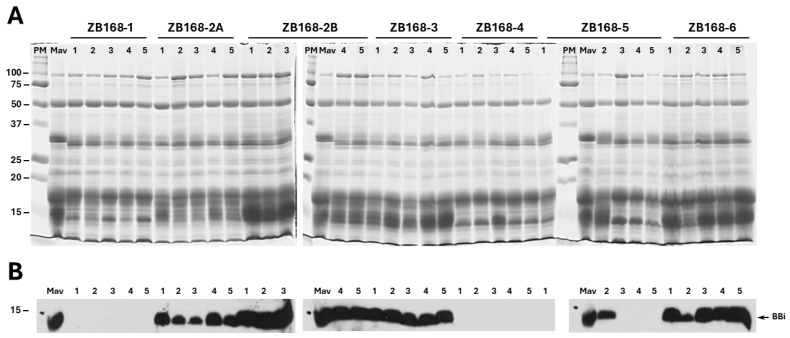
SDS-PAGE analysis of soybean seed proteins from RNAi-silenced BBi lines and control lines. (**A**) SDS-PAGE of 50% isopropanol-extracted seed proteins from T1 generation transgenic soybean plants. Seed proteins (30 µg/lane) from five individual seeds (lanes 1-5) for each of the seven independent transgenic events (ZB 168-1, ZB 168-2A, ZB 168-2B, ZB 168-4, ZB 168-5, and ZB 168-6) were separated on 15% SDS-PAGE gels and the proteins were visualized by staining with Coomassie Blue. (**B**) The proteins shown in panel A were electrophoretically transferred to a nitrocellulose membrane and incubated with polyclonal antibodies specific to soybean BBi antibodies. Immunoreactive proteins were detected using anti-rabbit immunoglobulin G (IgG)–horseradish peroxidase conjugate, following chemiluminescent detection. The names of the seven transgenic events are shown at the top of the figure. PM = protein markers; Mav = ‘Maverick’ (Mav., non-transgenic control). The numbers on the left side of the figure indicate the sizes of the protein markers in kilodaltons.

Although it has been shown that there are at least 13 *BBi* genes present in the soybean genome, transcriptome analysis has revealed that Glyma.16G208900 is abundantly expressed, while Glyma.14G117700 and Glyma.09G158700 are expressed at relatively low levels, in soybean seeds [22]. Interestingly, these seed-specific *BBi* genes share extensive sequence homology (Supplemental Figure S1) that enabled us to select conserved sequences for the simultaneous knockdown of seed-specific *BBi* genes. This was accomplished by RNAi, utilizing the Agrobacterium-mediated cotyledonary node transformation protocol, resulting in the generation of seven independent transgenic events. Seeds from these seven events were germinated and grown in a greenhouse to obtain T2 generation seeds. Seed proteins from five individual seeds, representing each of the seven transgenic events, were isolated and subjected to immunoblot analysis for the verification of the knockdown of BBi accumulation. We have previously shown that the extraction of soybean seed powder in 50% isopropanol resulted in the preferential accumulation of BBi [23]. Hence, we isolated 50% isopropanol-soluble seed proteins (Figure 1A) from these transgenic events and subjected them to Western blot analysis, utilizing BBi-specific polyclonal antibodies (Figure 1B). Our analysis revealed that the BBi accumulated during three independent transgenic events, although some differences in the accumulation levels were observed. However, in one event (ZB168-5), BBi accumulation was detected in one out of the five seeds examined. In two other transgenic events (ZB168-1 and ZB168-4), no proteins cross-reacting against the BBi antibodies were detected (Figure 1B).

**Figure 2:**
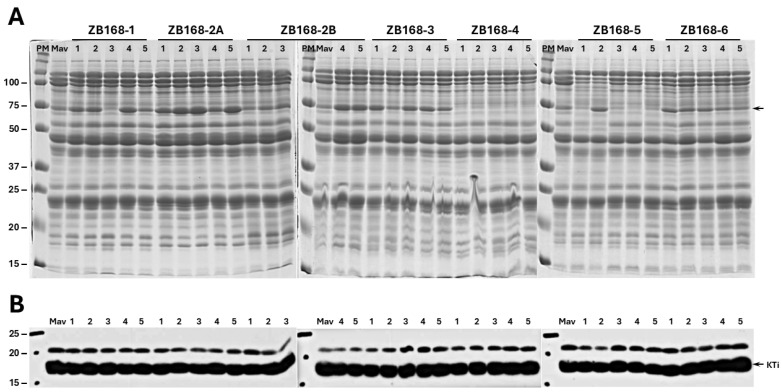
SDS-PAGE analysis of soybean seed proteins from RNAi-silenced BBi lines and control lines. (**A**) Sodium dodecyl sulfate polyacrylamide gel electrophoresis of total seed proteins from T1 generation transgenic soybean plants. Seed proteins (50 µg/lane) from five individual seeds for each of the seven independent transgenic events (ZB 168-1, ZB 168-2A, ZB 168-2B, ZB 168-4, ZB 168-5, and ZB 168-6) were separated on 13.5% SDS-PAGE gels and the proteins were visualized by staining with Coomassie Blue. (**B**) The proteins shown in panel A were electrophoretically transferred to a nitrocellulose membrane and incubated with antibodies specific to the soybean Kunitz trypsin inhibitor. Immunoreactive proteins were detected using anti-rabbit immunoglobulin G (IgG)–horseradish peroxidase conjugate, following chemiluminescent detection. The names of the seven transgenic events are shown at the top of the figure. PM = protein markers; Mav = ‘Maverick’ (Mav., non-transgenic control). The numbers on the left side of the figure indicate the sizes of the protein markers in kilodaltons. The arrow points to the β-subunit of β-conglycinin.

An earlier study has shown that a Kunitz trypsin inhibitor mutant (KTi3) over accumulated BBi to compensate for the loss of trypsin inhibitor activity [17]. To examine whether there is any reciprocal enhancement of the KTi due to the knockdown of the BBi, we performed Western blot analysis using the total seed proteins isolated from the dry seeds from the seven independent transgenic events (Figure 2A). Immunoblot blot analysis revealed uniform accumulation of the KTi protein in regard to all the transgenic events, even in regard to those events that failed to accumulate BBi (Figure 2B). An examination of the Coomassie Blue-stained total protein profile of the transgenic soybean events revealed significant differences in the accumulation of the β-subunit of β-conglycinin (Figure 2A, arrow).

### 2.2. Both Kunitz Trypsin Inhibitor and Bowman–Birk Protease Inhibitor Activities Are Reduced in BBi Knockdown Lines

**Figure 3:**
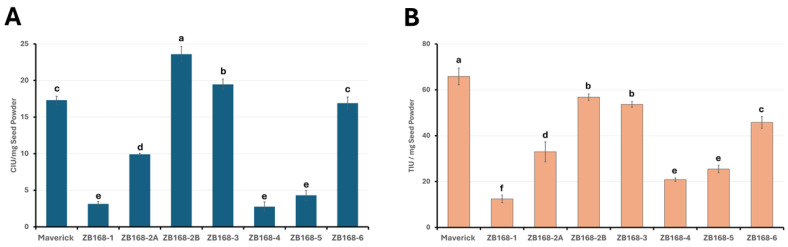
Analysis of chymotrypsin inhibitor activity (**A**) and trypsin inhibitor activity (**B**) in seeds from transgenic BBi-silenced and control plants. Chymotrypsin and trypsin inhibitor activities were measured at 37 °C, utilizing N-Succinyl-Ala-Ala-Pro-Phe p-nitroanilide and N-benzoyl-D,L-arginine 4-nitroanilide as substrates, respectively. Inhibitor activities are expressed as CIU (chymotrypsin units inhibited) and TIU (trypsin units inhibited) per milligram of seed powder. The error bars indicate the standard error of the mean (*n* = 3). Different letters on the top of each column indicate significant differences between the means.

The results of the immunoblot analysis revealed the absence of BBi accumulation in some of the RNAi-silenced BBi transgenic plants. To verify whether these plants were also compromised in regard to their protease inhibitor activity, we measured the chymotrypsin inhibitor activity of the RNAi-silenced plants and compared them to the levels found in wild-type soybean plants. The chymotrypsin inhibitor activity (CIU) was measured using N-Succinyl-Ala-Ala-Pro-Phe p-nitroanilide as a substrate, expressed as the CIU per mg of seed powder. The wild-type soybean seeds contained 17.2 CIU, while the BBi-mutated transgenic seeds showed activity ranging from 2.89 to 15.9 CIU. Out of the seven transgenic events examined, four exhibited drastic reductions, with a 2.8-6.0-fold decrease in the CIU compared to the wild type (Figure 3A). The chymotrypsin inhibitor activity in the seeds for four transgenic events (ZB168-1, ZB168-2A, ZB168-4, and ZB168-5) were significantly lower than the wild-type seeds. In regard to the transgenic events, ZB168-1 and ZB168-4, the chymotrypsin inhibitor activity was reduced by greater than 80% compared to the wild-type seeds. In regard to the other two transgenic events (ZB168-5 and ZB168-2A), the chymotrypsin inhibitor activity was reduced by 75% and 45%, respectively (Figure 3A). In contrast, the chymotrypsin inhibitor activity was not impacted in regard to transgenic event ZB168-5 6, while slightly elevated activity was detected in regard to transgenic event ZB168-2B and ZB168-3 compared to wild-type seeds (Figure 3A).

Since the soybean BBi can inhibit both chymotrypsin and trypsin, we also performed trypsin inhibitor measurements, using Nα-Benzoyl-L-arginine 4-nitroanilide hydrochloride as a substrate. The trypsin inhibitor activity was expressed as the TIU per mg of seed powder (Figure 3B). The wild-type soybean seeds averaged 57.25 TIU per mg of seed powder, while, in the BBi-silenced transgenic plants, the trypsin inhibitor activity ranged from 14.46 to 35.7 TIU (Figure 3B). Out of the seven transgenic events, four events, ZB168-1, ZB168-2A, ZB168-4, and ZB168-5, revealed significantly lower trypsin inhibitor activity compared to wild-type plants (Figure 3B). The trypsin inhibitor activity in other transgenic lines (events ZB168-2B, ZB168-3, and ZB168-6) was only marginally reduced compared to wild-type seeds (Figure 3B).

The seeds from three transgenic events (ZB168-1, ZB168-4, and ZB168-5) that failed to accumulate BBi proteins and showed significantly lower trypsin and chymotrypsin inhibitor activities were advanced to T3 and T4 generation. The absence of the BBi in the T3 and T4 generation was confirmed by immunoblot analysis, using BBi-specific polyclonal antibodies. For all further analysis, seeds from the T4 generation of ZB168-1 were employed.

### 2.3. Confocal Fluorescent Microscopy Reveals Drastic Reduction in the Accumulation of BBi in BBi Knockdown Seeds

**Figure 4:**
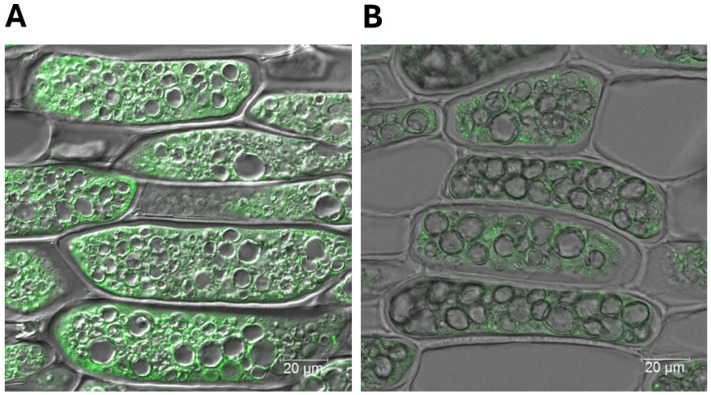
Immunofluorescence scanning confocal microscopy localization of Bowman–Birk protease inhibitor in soybean seeds. Thin sections of paraffin-embedded seeds of wild-type and BBi-silenced transgenic plants were incubated with soybean Bowman–Birk protease inhibitor antibodies and goat anti-rabbit Alexa Fluor Plus 488 and observed using scanning confocal microscopy. Note green fluorescence signals were mostly seen in the cell cytosol. (**A**) Wild type; and (**B**) ZB168-1.

Immunocytochemical localization of the BBi was performed by incubating thin sections of paraffin-embedded soybean seeds first with BBi-specific antibodies, followed by Alexa Fluor 488-labeled goat anti-rabbit antibodies. When thin sections of wild-type soybean seeds were examined under a scanning confocal microscope, bright green fluorescence was observed in all the parenchymatous cotyledonary cells (Figure 4A). Interestingly, fluorescence was primarily restricted to the cytoplasm. No specific localization of the BBi was detected in the protein storage vacuoles, which are the main storage organelles for the abundant seed storage proteins of soybeans. An examination of the thin sections of BBi knockdown soybean seeds revealed a drastic reduction in the intensity of green fluorescence in the parenchymatous cotyledonary cells (Figure 4B), indicating very low accumulation of BBi in the transgenic seeds.

### 2.4. RNAseq Analysis

We investigated whether the silencing of BBi genes resulted in any changes in the transcriptome of soybean seeds by performing RNA-seq at three development stages. The raw sequencing reads were trimmed to remove possible adapter sequences and nucleotides with poor quality, before being mapped to the Glycine_max_Wm82a4v1 reference genome map. This analysis revealed a mapping efficiency ranging between 62 and 63% for young, 82–91% for mid-stage, and 91–94% for late seed developmental stages. Using expression values from each sample, we constructed a sample–sample distance heatmap, showing the clustering of gene expression profiles between wild-type and BBi-silenced soybean seeds (ZB168-1) for all three developmental stages (Supplemental Figure S2). Principal component analysis (PCA) of the RNA-seq data revealed a high similarity between the three biological replicates at each of the three seed developmental stages.

#### 2.4.1. Impact of BBi Silencing on Soybean Transcriptome

**Figure 5:**
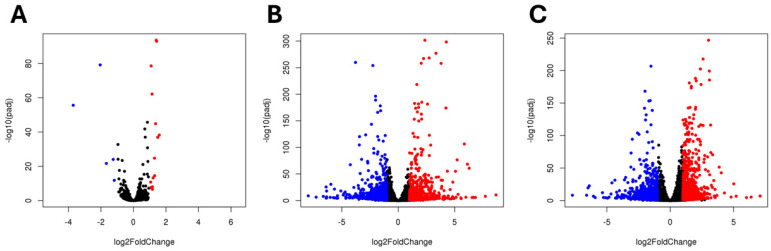
Volcano plot showing global transcriptional changes at three seed developmental stages ((**A**)-early, (**B**)-middle, and (**C**)-late). The log2 fold change of each gene is represented on the x-axis and the log10 of its adjusted *p*-value is on the y-axis. Genes with an adjusted *p*-value of less than 0.05 and a log2 fold change greater than 1 are indicated by red dots (upregulated genes). Genes with an adjusted *p*-value of less than 0.05 and a log fold change less than −1 are indicated by blue dots (downregulated genes).

Using DESseq2, a comparison of gene expression between the RNAi-derived BBi mutant (ZB168-1) and the wild type (‘Maverick’ soybean) was performed. For the visual identification of genes with large fold changes that are statistically significant, we constructed Volcano plots (Figure 5). Genes with an adjusted *p*-value of less than 0.05 and a log2 fold change greater than 1 are indicated by red dots. Similarly, genes with an adjusted *p*-value of less than 0.05 and a log2 fold change greater than −1 are indicated by blue dots. In these plots, the most upregulated genes (red dots) in the RNAi-derived BBi mutant are shown on the right, while the most downregulated genes (blue dots) are on the left side of the figure. During the early stages of seed development, 318 genes were found to be downregulated and 167 genes were upregulated. At early-stage seed development, a limited number of genes in the RNAi-derived BBi mutant were either upregulated or downregulated. The upregulated genes included five genes (*Glyma.15G119800*, *Glyma.15G119700*, *Glyma.15G199600*, *Glyma.20G148200*, and *Glyma.18G157800*) with a log fold change greater than 1. Similarly, three genes (*Glyma.16G208900*, *Glyma.08G087100*, and *Glyma.19G178100*) were downregulated (Figure 5A). At the mid-stage of seed development, there were 2539 DEGs, out of which 1119 genes were upregulated and 1420 genes were downregulated (Figure 5B). During the later stages of seed development, a greater number of DEGs between the wild-type and CRISPR–BBi knockdown lines were observed (Figure 5C). At this stage, there were 3347 DEGs, out of which 1284 genes were upregulated, and 2063 genes were downregulated (Figure 5C). Since the number of DEGs was relatively large, we applied a stringent filtering threshold to obtain a list of highly correlated DEGs. By applying a stringent filtering threshold of |log2FC| ≥ 3 and *p*-value < 0.01, genes located at the extreme ends of the volcano plot were obtained and are listed in Supplementary Table S2.

#### 2.4.2. Expression Profile of Differentially Expressed Genes in Wild-Type and BBi-Silenced Soybean Seeds

**Figure 6:**
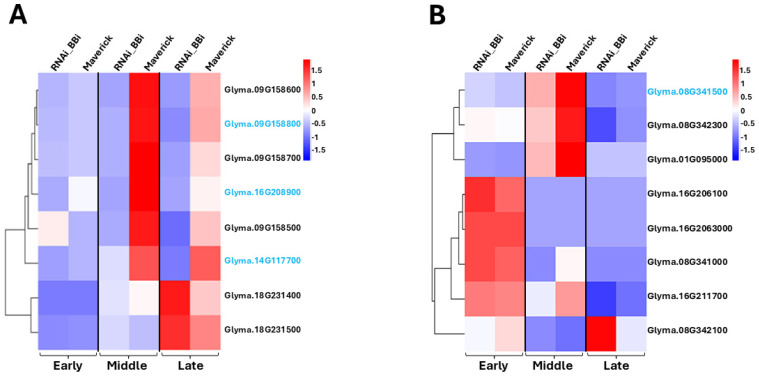
Heat map displaying the expression profile of BBi genes (**A**) and KTi genes (**B**) across three seed developmental stages (early, middle, and late). The most abundantly expressed KTi and BBi genes are shown in blue font. Maverick = wild type; RNAi_BBi = transgenic event ZB168-1.

The soybean genome contains at least 13 different BBi genes, out of which only a few of them are expressed in appreciable levels in soybean seeds [11,22]. Transcriptomic analysis has shown that *Glyma.16g208900* encodes the most abundantly expressed BBi gene in developing soybean seeds, followed by *Glyma.14G117700*. The other BBi genes that are expressed, albeit at very low levels, are *Glyma.09G158700*, *Glyma.09G158600*, *Glyma.09G158800*, and *Glyma.09G158500*, respectively. We examined the expression pattern of these six seed-expressed BBi genes in wild-type and BBi-silenced seeds (Figure 6A). All the BBi genes were expressed in wild-type soybean seeds, with the highest levels of expression detected at the mid-stage of development. As was reported earlier, *Glyma.16g208900* was the most abundantly expressed BBi gene in wild-type soybean seeds. However, the expression of this gene was not detected in BBi-silenced transgenic seeds. Interestingly, low levels of expression of other BBi genes (*Glyma.14G117700*, *Glyma.09G158700*, *Glyma.09G158600*, *Glyma.09G158800*, and *Glyma.09G158500*) was detected in BBi-silenced transgenic seeds, although their expression levels were significantly reduced compared to the wild-type seeds (Figure 6A).

Our studies have shown that the BBi-silenced soybean seeds contained reduced levels of trypsin inhibitor activity compared to that of the wild-type seeds. Hence, we wanted to examine whether there are any differences in the expression levels of KTi genes in wild-type and BBi-silenced soybean seeds. There are three KTi genes (KTi1, KTi2, and KTi3) in soybeans, with KTi3 being the most abundantly expressed gene in soybean seeds [9]. A comparison of the expression levels of the KTi genes revealed that the expression of all three KTi genes were almost similar at the early seed developmental stage between the wild-type and BBi-silenced soybean seeds (Figure 6B). The expression of the KTi2 gene peaked during the early developmental stage, declined at the mid-stage, and was undetectable by the late stage. In contrast, the expressions of KTi1 and KTi3 were abundant at the mid-stage and declined at the later stages. Although a significant decrease in KTi1 and KTi3 expression was observed in BBi-silenced soybean seeds compared to the wild type at the mid-stage of development, their expression levels were similar at the later stages of seed development (Figure 6B).

### 2.5. BBi Disruption Has a Minimal Impact on Seed Quality Traits in Soybeans

**Figure 7:**
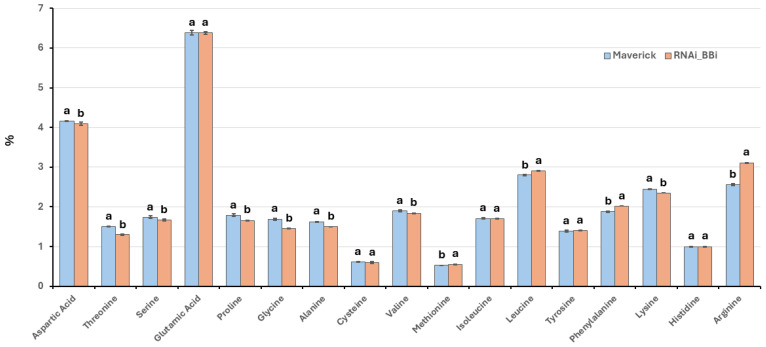
HPLC measurement of amino acid content of wild-type (Maverick) and BBi-silenced soybean (ZB168-1) seeds. Error bars represent the standard error of the mean, calculated from three biological replicates. Different lowercase letters in the same amino acid column indicate significant differences among the means (*p* < 0.05).

The BBi is a sulfur-rich protein, characterized by the presence of multiple cysteine residues [24,25]. To investigate whether silencing BBi genes affects the sulfur-containing amino acid content, we conducted amino acid profiling, using high-performance liquid chromatography (Figure 7). Overall, the amino acid compositions were largely consistent across the wild-type and knockdown seeds. Nevertheless, a one-way ANOVA, followed by Tukey’s HSD post hoc test (*p* < 0.05), indicated significant differences in the levels of several individual amino acids (Figure 7). Notably, the cysteine levels remained statistically unchanged between the two genotypes. Interestingly, the methionine content showed a slight increase in the BBi knockdown line (0.55%) compared to the wild type (0.53%). We also measured the protein and oil content of the seeds using near-infrared reflectance (NIR) spectroscopy. The protein and oil contents of the wild-type soybean or Maverick were 40.49 ± 0.42% and 20.01 ± 0.48%, respectively. Similarly, the protein and oil contents of BBi-silenced soybean seeds were also 40.49 ± 0.18% and 20.01 ± 0.12%, respectively.

Our investigation indicates that despite the pronounced downregulation of BBi expression, there were no appreciable differences in the seed protein, oil content, or sulfur amino acid composition. These findings suggest that BBi gene disruption exerts minimal influence on seed nutritional quality and composition in soybean.

### 2.6. Protein Digestibility Is Enhanced in BBi Knockdown Soybean Line

**Figure 8:**
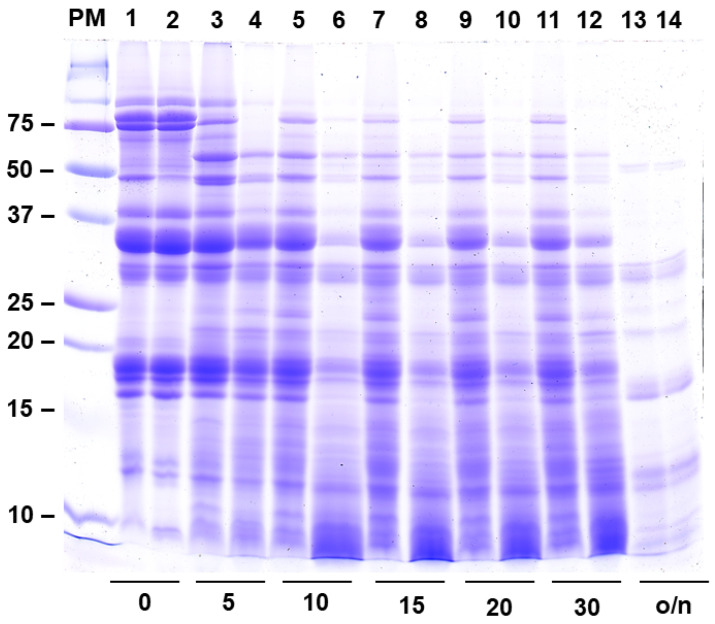
In vitro protein digestibility of soybean seed proteins. Seed proteins isolated from wild-type (lanes 1, 3, 5, 7, 9, 11, and 13) and BBi-silenced soybean (ZB168-1) seeds (lanes 2, 4, 6, 8, 10, 12, and 14) were digested with trypsin and chymotrypsin. Aliquot of samples were removed at 0, 5, 10, 15, 20, 30 min, and overnight (o/n) after incubation, and separated on 13.5% SDS-PAGE gels and visualized using Coomassie Brilliant Blue staining. Note the rapid digestion of high-molecular-weight proteins and the accumulation of oligopeptides (<10 kDa) in lanes corresponding to BBi-silenced protein extracts. The numbers at the bottom of the figure refer to minutes; o/n = overnight. The numbers on the left side of the figure indicate the sizes of the protein markers in kilodaltons.

Since the BBi inhibits both of the digestive enzymes, trypsin and chymotrypsin, we wanted to examine whether the reduction in the BBi activity had any effect on protein digestibility. An examination of the SDS-PAGE-resolved protein profiles at different times of digestion with trypsin and chymotrypsin revealed that the seed proteins in the BBi knockdown lines were much more rapidly digested compared to that of wild-type seed proteins (Figure 8). The increased digestibility of seed proteins can be observed within 5 min after incubation with trypsin and chymotrypsin. The appearance of low molecular peptides at the bottom of the SDS-PAGE gels indicates that the lack of BBi has resulted in the increased susceptibility of soybean seed proteins to trypsin and chymotrypsin (Figure 8).

## 3. Discussion

Our study provides experimental evidence demonstrating that RNAi-generated transgenic soybean lines exhibit significantly reduced protease inhibitor activity. *BBi* knockdown lines show not only a drastic reduction in chymotrypsin inhibitor activity, but also in trypsin inhibitor activity. This outcome is expected, as BBi inhibits both trypsin and chymotrypsin, two critical digestive enzymes. Western blot analysis confirmed that the *BBi* knockdown lines failed to accumulate this protein in their seeds, while transcriptomic analysis revealed a substantial downregulation of most seed-specific *BBi* genes in the knockdown lines. However, the suppression of the most abundant seed-specific *BBi* genes did not eliminate chymotrypsin inhibitor activity. All the transgenic soybean lines generated in this study exhibited residual chymotrypsin inhibitor activity, albeit at much lower levels than wild-type soybeans. We speculate that the residual chymotrypsin inhibitor activity may be due to the upregulation of minor seed-specific *BBi* genes. Supporting this hypothesis, our transcriptomic analysis revealed that two *BBi* genes, Glyma.18G231400 and Glyma.18G231500, which are expressed at extremely low levels in wild-type seeds, exhibited increased expression in the *BBi* knockdown line. This upregulation could account for the residual chymotrypsin inhibitor activity observed in the *BBi* knockdown lines.

Previous studies have demonstrated that gene editing technologies can be effectively utilized to reduce the activity of Kunitz trypsin inhibitors (KTIs) and Bowman–Birk inhibitors (BBis) in soybean seeds [21,26,27]. Specifically, CRISPR/Cas9-mediated genome editing was employed to introduce small insertions or deletions within the open reading frames of the *kti1* and *kti3* alleles in the soybean cultivar, Williams 82 [26]. The resulting *kti1/kti3* double mutants exhibited a marked reduction in KTi and overall trypsin inhibitor (TI) activity compared to wild-type seeds. Importantly, these genetic modifications did not adversely affect plant growth or the days to maturity under greenhouse conditions [26]. In parallel, recent research targeting *BBi* genes using CRISPR/Cas9 revealed dramatic reductions in total trypsin inhibition and chymotrypsin inhibition relative to the wild-type controls [21,27]. More importantly, the *BBi* knockdown lines also showed potential for improved processing efficiency, as they required less thermal treatment to inactivate residual protease inhibitors [27], an advantage that could reduce energy costs and preserve protein quality during soybean meal production. Collectively, these findings underscore the promise of genome editing as a powerful strategy to minimize antinutritional factors in soybean seeds, thereby enhancing the nutritional value of soybean-derived food and feed products.

Earlier ultrastructural studies of soybean cotyledons and embryonic axes have shown that the Bowman–Birk inhibitor (BBi) is localized in protein bodies, the nucleus, and, to a lesser extent, the cytoplasm [28]. However, our confocal fluorescent microscopy study clearly demonstrates that the BBi is exclusively localized in the cytoplasm and not in protein storage vacuoles. Protease inhibitors, including the Kunitz trypsin inhibitor (KTi) and BBi, belong to the 2S albumin fraction, but their subcellular localization in legumes remains incompletely understood. In mung beans, fluorescent antibody studies have shown that protease inhibitors are primarily associated with the cytoplasm [29]. Similarly, in *Adenanthera pavonina*, immunocytochemical localization studies have confirmed that protease inhibitors are predominantly found in the cytoplasm, rather than in protein bodies or protein storage vacuoles [30]. Based on these findings, together with our own observations, we conclude that protease inhibitors are primarily localized in the cytoplasm of legume seeds.

Soybeans, like other legumes, contain relatively low levels of sulfur-containing amino acids, such as cysteine and methionine [31]. Bowman–Birk inhibitors (BBi) serve as a sulfur amino acid reserve in soybean seeds, as they contain seven disulfide bonds. The amino acid composition of the mature BBi protein, which consists of 71 amino acids, includes 14 cysteine residues [24,25]. Thus, eliminating BBi accumulation could theoretically lead to a drastic reduction in the overall cysteine content of the seeds. However, amino acid analysis of *BBi*-silenced lines reveals no decrease in the cysteine content of seed proteins compared to wild-type soybeans. This observation suggests that, in the absence of BBi, available cysteine may be incorporated into other cysteine-rich proteins. Previous studies have shown that proteomic rebalancing occurs when the accumulation of abundant seed proteins is disrupted [32]. A more in-depth proteomic analysis of seed proteins could clarify whether proteomic rebalancing takes place in *BBi*-silenced lines.

Proteinase inhibitors are widely recognized for their critical role in defending plants against a broad range of pathogens and insect pests [33,34,35,36]. Given that the BBi knockdown lines exhibit significantly reduced levels of these inhibitors, it is essential to investigate whether this reduction renders the plants more susceptible to biotic stress. To address this, comprehensive field evaluations will be necessary to assess the performance of the BBi knockdown lines under natural pathogen and pest pressure. These trials should determine whether the reduction in BBi levels leads to agronomic compromise, including yield loss, reduced stress tolerance, or other undesirable traits.

A recent study [37] compared the protein digestibility of seed proteins in a triple-null soybean mutant, lacking the Kunitz trypsin inhibitor (KTi), agglutinin, and the P34 allergen, to that of the wild type, using an in vitro protein digestibility assay. Contrary to our findings, they reported no significant improvement in the protein digestibility of the triple mutant compared to the corresponding control lines. This discrepancy may be attributed to the presence of the Bowman–Birk inhibitor (BBi) in the triple mutant, despite the absence of the KTi. In soybean seeds, the total protease inhibitor activity results from the combined effects of the KTi and BBi. Previous research has shown that the KTi mutant retains a substantial amount of trypsin inhibitor activity [17], whereas the BBi mutant exhibits a much more pronounced reduction in both trypsin and chymotrypsin inhibitor activity [21]. These findings suggest that the BBi is the predominant contributor to protease inhibitor activity in soybean seeds. Therefore, it is reasonable to expect that a soybean BBi mutant would demonstrate improved protein digestibility compared to a KTi mutant.

Soybeans have long been regarded as the “gold standard” against which other protein sources are compared. Their high protein content, coupled with a well-balanced amino acid profile, makes soybeans an ideal protein ingredient in animal feed. However, the full nutritional potential of soybeans is hindered by the presence of several antinutritional factors (ANFs), such as lectins, trypsin inhibitors, and chymotrypsin inhibitors. These ANFs can significantly impair protein digestion and reduce nutrient absorption in the intestine [38]. In our study, we developed *BBi* knockdown soybean lines that show great promise for improving the formulation of animal feed. These lines contain significantly reduced levels of trypsin and chymotrypsin inhibitors, two key ANFs that typically require extensive heat treatment in order to be neutralized. By reducing the levels of these protease inhibitors, the *BBi* knockdown lines offer several advantages. They are likely to require minimal heat processing, translating into substantial cost savings during production. Additionally, the absence of protease inhibitors enhances protein digestibility, thereby improving nutrient utilization and boosting animal performance. The results of our in vitro protein digestion analysis further underscore the benefits of the *BBi* knockdown lines. Proteins derived from these soybeans are broken down more efficiently into lower molecular weight oligopeptides compared to those from wild-type soybeans. This improved digestibility highlights the potential of *BBi* knockdown soybeans as a superior ingredient in animal feed, offering both economic and nutritional benefits.

Efforts to reduce or eliminate protease inhibitors in soybean seeds have long been a key objective for soybean breeders. Previously, we developed a soybean germplasm with the lowest reported levels of trypsin inhibitor in conventional soybeans [17]. This was achieved through traditional crossing of two soybean PI lines lacking KTi-3 and KTi-1. However, the resulting progeny still exhibited significant trypsin inhibitor activity, due to the presence of Bowman–Birk inhibitor (BBi) proteins. To develop soybean lines completely devoid of protease inhibitors, both the KTi and BBi must be eliminated. Using RNAi technology, we have successfully generated soybean lines with nearly undetectable levels of the BBi in seeds. Moreover, these transgenic lines show significantly reduced trypsin and chymotrypsin inhibitor activities. We are now optimally positioned to combine these two traits, eliminating both the KTi and BBi, into a single soybean line. The creation of soybean lines lacking both KTi and BBi will enhance the seed value for farmers and enable the use of raw soybean meal in swine, poultry, and other animal feed.

## 4. Materials and Methods

### 4.1. Chemicals

Most chemicals and reagents used in this study were of analytical grade. Trypsin, chymotrypsin, β-mercaptoethanol, Nα-Benzoyl-DL-arginine p-nitroanilide hydrochloride, and N-Succinyl-Ala-Ala-Pro-Phe p-nitroanilide were purchased from Sigma-Aldrich (St. Louis, MO, USA). Acrylamide, bis acrylamide, SDS, TEMED, and ammonium persulfate were purchased from GE healthcare (Piscataway, NJ, USA).

### 4.2. Construction of RNAi Cassettes to Downregulate Bowman–Birk Inhibitor Genes and for Soybean Transformation

To simultaneously suppress the expression of Bowman–Birk inhibitors (BBi), we constructed an RNAi cassette, using the following procedure. First, the *BBi* promoter region (Glyma.16G208900) was amplified from the soybean cultivar Maverick genomic DNA, utilizing the primer pair 5′H3Xh16G_H_Promoter (5′-AAGCTTCTCGAGATTCAACGGTAAATTTATTG-3′), which contains HindIII and XhoI restriction sites, and 16G_H_PromoterBH (5′-GGATCCGTTGTTCTTCAAACTCATCTTTATTAATTG-3′), which contains a BamHI restriction site. The amplified *BBi* promoter was cloned into pGEM-T Easy vector and designated as pGBBipro. The promoter region was then excised using HindIII and BamHI and cloned into the corresponding restriction site of the pZPlapha′P binary vector [21], resulting in pZBBipro.

The coding region of the soybean Bowman–Birk inhibitor (*BBi*) was amplified from soybean seed total RNA, using primers 5′-CTCTTCACAGCAAAAACAATTAAT-3′ and 5′-CCATTTGAGAGAGCTATTAGTTTTTC-3′. The amplified PCR product was cloned into the pGEM-T Easy vector and designated as pGBBi16G. The sense and antisense *BBi* fragments were amplified from pGBBi16G, using the primer pair SNd16G_H_RNAi (5′-GTCGACCATATGAGTTTGAAGAACAAC-3′), with introduced SalI and NdeI restriction sites, and 16G_H168_RNAi_XhR (5′-GATATCCTCGAGTGTGCACATGCAGAGATCAC-3′), with introduced EcoRV and XhoI restriction sites. The amplified 168 bp sense and antisense fragments were cloned into pGEM-T Easy vector and designated as pGBBi168RNAi.

The sense fragment was excised from pGBBi168RNAi using NdeI and XhoI, then cloned into the PHK vector, resulting in PHK168iA. The antisense fragment was excised from pGBBi168RNAi using EcoRV and SalI, then cloned into PHK168iA, generating PHK168iAB. Following digestion with XbaI, the entire BBi hairpin sequence was cloned into the corresponding restriction site of the pZBBipro binary vector, designated as pZBBi_168. This final vector (Supplementary Figure S3) places the *BBi* hairpins under the regulatory control of the *BBi* promoter, the α′-subunit of the β-conglycinin promoter, and the terminator of the potato proteinase inhibitor gene (PinII). Additionally, the vector contains the barcoding region under the regulatory control of the cauliflower mosaic virus (CaMV) 35S promoter and the 3′ region of the nopaline synthase gene. The RNAi construct was mobilized into a disarmed *Agrobacterium tumefaciens* strain, EHA105, via heat-shock transformation.

The soybean transformation was performed, following the protocol described previously [21]. ‘Maverick’ soybean (Reg. no. CV-372, PI 598124), which was developed by the Missouri and Illinois Agricultural Experiment Stations at the Universities of Missouri and Illinois, respectively, was used for plant transformation. Putative transgenic soybean plants were identified by a leaf paginating assay, using 100 mg of L−1 glufosinate ammonium solution on leaves at three stages [39]. Herbicide-resistant plants were then cultivated in greenhouses under controlled conditions, maintaining a 16 h day at 28 °C and an 8 h night at 24 °C.

### 4.3. Protein Extraction and 1-D Electrophoresis

The total soybean seed protein extraction and subsequent separation using 1-D SDS-PAGE were conducted as previously described [21]. Briefly, dry soybean seeds were ground into a fine powder, using a mortar and pestle. Ten mg of seed powder was transferred into 1.5 mL plastic tubes, to which 1 mL of SDS sample buffer (60 mM Tris-HCl, pH 6.8, 2% SDS, 10% glycerol, and 5% 2-mercaptoethanol) was added. The tubes were vortexed for 10 min at room temperature, then heated to 100 °C for 5 min, followed by centrifugation at 15,800× *g* for 5 min. The supernatant, representing the total seed protein fraction, was resolved using 13.5% SDS-PAGE gels in a Hoefer SE 260 minigel apparatus (Amersham Biosciences, Piscataway, NJ, USA). Electrophoretic separation was carried out at a constant current of 20 mA per gel. The separated proteins were visualized using Coomassie Blue R-250 solution (0.3% Coomassie Brilliant Blue R-250, 45% methanol, and 10% glacial acetic acid).

### 4.4. Immunoblot Analysis

The proteins were extracted from the wild-type soybean cultivar ‘Maverick’ and T1:2 seeds of the *BBi*-mutated lines were separated using 13.5% SDS-PAGE gels. These proteins were then transferred to nitrocellulose membranes and incubated with TBS (10 mM Tris-HCl, pH 7.5, 500 mM NaCl), supplemented with 5% non-fat dry milk for 1 h at room temperature. After incubation, the nitrocellulose membranes were washed three times with TBST (15 min each) and incubated overnight with antibodies at a 1:10,000 dilution, specifically raised against either purified soybean Kunitz trypsin or Bowman–Birk protease inhibitors [23]. The specifically bound primary antibodies were detected using anti-rabbit IgG–horseradish peroxidase conjugates and the SuperSignal West Pico kit (Pierce, Rockford, IL, USA).

### 4.5. Trypsin and Chymotrypsin Inhibitor Assay

To measure the trypsin inhibitor activity in various *BBi*-mutated CRISPR/CAS9 lines, the methodology described by Kim and Krishnan, 2024 [21], was followed. In brief, 20 mg of dry soybean seed powder was placed into a 2 mL Eppendorf tube, then 1 mL of 10 mM NaOH solution was added. This mixture was vigorously agitated, using a vortex mixer, for 10 min at room temperature. The resulting slurry was clarified through centrifugation at 16,000× *g* for 10 min, and the clear supernatant was used to determine the KTi activity. The trypsin inhibitor activity was quantified as the trypsin units inhibited (TUI) per mg of the sample, with the results presented as the mean ± SD from three biological replicates.

To assess the chymotrypsin inhibitor activity, soybean seed extracts were used, with N-Succinyl-Ala-Ala-Pro-Phe p-nitroanilide (AAPF) serving as the substrate. The soybean seed extract, which inhibited 40 to 60% of chymotrypsin, was added into a 1.5 mL Eppendorf tube, containing 900 µL of assay buffer (100 mM-Tris-HCL, pH 8.0), 8 µL of α-chymotrypsin (Sigma-Aldrich Company), dissolved in 1 mM HCl solution (0.1 mg/mL), and 8 to 10 µL of seed extract. This mixture was incubated at 37 °C for 5 min. Subsequently, 80 µL of AAPF (1 mg/mL) was added, and the incubation was continued for an additional 10 min at 37 °C. The assay was terminated by adding 500 µL of 30% acetic acid. Absorbance at 410 nm was measured, and the chymotrypsin inhibitor units were calculated based on the reduction in absorbance at 410 nm by 0.1 optical density. The results were expressed as the mean ± SD from three biological replicates. The data from the trypsin and chymotrypsin inhibitor assays were visualized and compared using the JMP statistical software version 16.0 (SAS Institute Inc., Cary, NC, USA). A one-way ANOVA was performed, and significant differences (α = 0.05) between the means were determined using the Tukey–Kramer HSD test.

### 4.6. RNA-Seq Analysis

The total RNA was isolated from developing seeds of the BBi mutant (ZB168-1) and the wild-type soybean cultivar ‘Maverick’ at three distinct reproductive stages: early (R5), middle (R6), and late (R7). RNA extraction was performed using the TRIzol^®^ Reagent (Thermo Fisher Scientific, Waltham, MA, USA). For each developmental stage, three biological replicates were included. The total RNA was treated with DNase I (Invitrogen, Carlsbad, CA, USA) to remove any contaminating genomic DNA. The RNAseq libraries were prepared by Azenta Life Sciences (Burlington, MA, USA). The process involved the conversion of single-stranded RNA into double-stranded complementary DNA (cDNA), with the addition of sequencing adapters and barcodes, which are subsequently analyzed using next-generation sequencing (NGS). Data analysis was conducted, and the identification of differentially expressed genes (DEGs) was determined.

### 4.7. Confocal Immunofluorescence Microscopy Localization of Bowman–Birk Protease Inhibitor

Soybean seeds soaked in water were cut into several small cubes, using a razor blade. The dissected seed samples were immediately fixed, as previously described [6], in a solution containing 2% paraformaldehyde and 2% glutaraldehyde, in 100 mM cacodylate buffer, pH 7.2. After fixation, the samples were dehydrated through the use of a graded ethanol series (80–95–100%) and embedded in paraffin. The paraffin-embedded samples were then processed for confocal immunofluorescence microscopy. Immunohistochemical localization was conducted on paraffin-embedded sections, utilizing antibodies against Bowman–Birk protease inhibitor peptides, raised in rabbits. Five micrometer sections of paraffin-embedded soybean cotyledons were mounted onto X-tra Plus microscope slides (Leica, Richmond, IL, USA), de-waxed in xylene, rehydrated through the use of graded ethanol concentrations, and finally in water. The sections were incubated for 60 min at room temperature, with a 1:200 dilution of the soybean BBi antibody, followed by a 30 min incubation with Alexa Fluor 488 Plus-conjugated goat anti-rabbit secondary antibody (Invitrogen/Thermo Fisher, Waltham, MA, USA), diluted 1:500. The sections were then cover slipped with a mounting medium containing an antifade reagent and observed under a Leica SP8 laser scanning confocal microscope (Leica Microsystems, Buffalo Grove, IL, USA), with a 20×/NA 0.7 objective, using a 495 nm excitation laser line and a 505–550 nm bandpass.

### 4.8. Protein, Oil, and Amino Acid Analyses

The protein and oil content were determined using near-infrared spectroscopy (FOSS North America, Eden Prairie, MN, USA). The amino acid analysis of the wild-type (Maverick) and *BBi* knockdown line (ZB168-1) was performed at the University of Missouri Agriculture Experiment Station Chemical Laboratories, University of Missouri. The amino acids were separated using a Beckman 6300 Amino Acid Analyzer (Beckman Coulter Life Sciences, Indianapolis, IN, USA), equipped with a high-performance, cation exchange resin column. The results are reported as the mean ± standard deviation from three biological replicates.

### 4.9. In Vitro Protein Digestibility Assay

Seed proteins from the wild-type (Maverick) and *BBi* knockdown line (ZB168-1) were isolated by extracting 30 mg of dry soybean seed powder with 1 mL of 10 mM Tris-HCl, pH 7.5, in a rotary shaker, for 20 min at room temperature. The slurry was centrifuged at 14,000 rpm for 3 min and the resulting supernatant was saved in separate tubes. To this supernatant, 10 µL of trypsin (10 mg/mL) and 10 µL of chymotrypsin (10 mg/mL) stock solution was added and incubated in rotary shaker, maintained at 37 °C. At intervals of 0, 5, 15, 60, and 120 min, 50 µL of protein solution was removed. To this 10 µL of 6× SDS Laemmli sample buffer was added, mixed thoroughly and placed in a boiling water bath for 5 min. The protein samples were resolved on 13.5% SDS-PAGE gels and the separated proteins were visualized by Coomassie Brilliant Blue staining.

## Data Availability

All the data supporting the findings of this study are provided in the manuscript and Supplementary Information.

## Supplementary Materials

The following supporting information can be downloaded at: https://www.mdpi.com/article/10.3390/ijms26146943/s1.
