# Supplementary material for: Seed-Specific Silencing of Abundantly Expressed Soybean Bowman–Birk Protease Inhibitor Genes by RNAi Lowers Trypsin and Chymotrypsin Inhibitor Activities and Enhances Protein Digestibility
Source: Int J Mol Sci. 2025 Jul 19;26(14):6943. doi: 10.3390/ijms26146943 (PMC12294982; doi:10.3390/ijms26146943)
Supplement: Supplementary file 1 [file ijms-26-06943-s001.zip › ijms-3728768-supplementary.pdf]

Supplemental Materials

Seed specific silencing of the abundantly expressed soybean Bown-Birk protease inhibitor genes by RNAi lowers the trypsin and chymotrypsin inhibitor activities and enhances the protein digestibility

Wonseok Kim [1], Sunhyung Kim [1], Hari B. Krishnan [1,2,*]

[1] Division of Plant Science and Technology, University of Missouri, Columbia, MO 65211, USA; wonseokk@missouri.edu (W.-S.K.); jeongs@missouri.edu (S.K.)
[2] Plant Genetics Research Unit, US Department of Agriculture-Agricultural Research Service, Columbia, MO 65211, USA
* Correspondence: hari.b.krishnan@usda.gov; Tel.: +1-573-882-8151

Supplemental Figure S1. Nucleotide sequence alignment of various BBi genes. Forward and reverse arrows indicate the primer binding sites selected from the BBi gene (Glyma.16G208900) that were used to amplify a 168-bp fragment, highlighted within the boxed region. * indicates identical bases; arrow indicates primer sequences; adenine is shown in red font; guanine, cytosine and thymine are shown in green font.

```
Glyma.09G158500.1      ATGGGTTTGAAGAACAACATGGTGGTGCTAAAGGTGTGTTTGGTGCTACTTTTCCTTGTG    60
Glyma.09G158900.2      ATGGGTTTGAAGAACAACATGGTGGTGCTAAAGGTGTGTTTGGTGCTACTTTTCCTTGTG    60
Glyma.14G117700.1      ATGGGTTTGAAGAACAACATGGTGGTGCTAAAGGTGTGTTTGGTGCTACTTTTCCTTGTG    60
Glyma.16G208900.1      ATGAGTTTGAAGAACAACATGGTGGTGCTAAAGGTGTGTTTGTTGCTTCTTTTCCTTGTG    60
Glyma.09G158600.1      ATGGGTTTGAAGAACAACATGGTGGTGCTAAAGGTGTGTTTCTTGGTTCTTTTCCTTGTG    60
Glyma.09G158700.1      ATGGGTTTGAAGAACAACATGGTGGTGCTAAAGGTGTGTTTCTTGGTTCTTTTCCTTGTG    60
                       *** *************************************  ** * ************

Glyma.09G158500.1      GGGGGTACTACTAGTGCCAACTTGAGGCTGAGTAAGCTTGGCCTGCTCATGAAAAGTGAT    120
Glyma.09G158900.2      GGGGGTACTACTAGTGCCAACTTGAGGCTGAGTAAGCTTGGCCTGCTCATGAAAAGTGAT    120
Glyma.14G117700.1      GGGGGTACTACTAGTGCCAGCTTGAGGCTGAGTGAGCTTGGCCTGCTCATGAAAAGTGAT    120
Glyma.16G208900.1      GGGGTTACAGCTGCACGCATGGAA---------------CTGAGCTTCTTCAAAAGTGAT    105
Glyma.09G158600.1      GGGGTTACTAATGCACGCATGGAA---------------CTGAACCTCTTCAAAAGTGAT    105
Glyma.09G158700.1      GGGGTTACTAATGCACGCATGGAA---------------CTGAACCTCTTCAAAAGTGAT    105
                       **** ***   *     **                           ** * *********

Glyma.09G158500.1      CATCATCAACACTCAAATGATGATGAGTCTTCAAAACCATGCTGTGATCAATGCGCATGC    180
Glyma.09G158900.2      CATCATCAACACTCAAATGATGATGAGTCTTCAAAACCATGCTGTGATCAATGCGCATGC    180
Glyma.14G117700.1      CATCATCAACACTCAAATGATGATGACTCTTCAAAACCATGCTGTGATCAATGCGCATGC    180
Glyma.16G208900.1      CAGTCATCAAGTTATGATGATGATGAGTATTCAAAACCATGCTGTGATCTCTGCATGTGC    165
Glyma.09G158600.1      CACTCATCAAGTGAT------GATGAGTCTTCAAAACCATGCTGTGATCTCTGCATGTGC    159
Glyma.09G158700.1      AACTCATCAAGTGAT------GATGAGTCTTCAAAACCATGCTGTGATCTCTGCATGTGC    159
                        *      *            ***** * ********************  ***   ***

Glyma.09G158500.1      ACAAAGTCAAACCCTCCTCAATGCCGCTGTTCAGATATGAGGCTGAATTCGTGCCATTCA    240
Glyma.09G158900.2      ACAAAGTCAAACCCTCCTCAATGCCGCTGTTCAGATATGAGGCTGAATTCGTGCCATTCA    240
Glyma.14G117700.1      ACAAAGTCAAACCCTCCTCAATGCCGCTGTTCAGATATGAGGCTGAATTCGTGCCATTCA    240
Glyma.16G208900.1      ACACGCTCAATGCCTCCTCAATGCAGCTGTGAAGATATTAGGCTGAATTCATGCCACTCA    225
Glyma.09G158600.1      ACAGCCTCAATGCCACCTCAATGCCATTGTGCAGATATTAGGTTGAATTCATGTCACTCA    219
Glyma.09G158700.1      ACAGCCTCAATGCCACCTCAATGCCATTGTGCAGATATTAGGTTGAATTCATGTCACTCA    219
                       ***   ****  ** *********   ***  ****** *** ******* ** ** ***

Glyma.09G158500.1      GCTTGCAAATCTTGTATTTGCGCATTATCGTATCCTGCACAGTGTTTTTGTGTTGACATA    300
Glyma.09G158900.2      GCTTGCAAATCTTGTATTTGCGCATTATCGTATCCTGCACAGTGTTTTTGTGTTGACATA    300
Glyma.14G117700.1      GCTTGCAAATCTTGTATTTGCGCATTATCGTATCCTGCACAGTGTTTTTGTGTTGACATA    300
Glyma.16G208900.1      GATTGTAAGAGCTGTATGTGCACACGCTCACAGCCAGGACAGTGTCGTTGTCTTGACACC    285
Glyma.09G158600.1      GCTTGTGATCGCTGTGCGTGCACACGCTCGATGCCAGGCCAGTGTCGTTGCCTTGACACC    279
Glyma.09G158700.1      GCTTGTGATCGCTGTGCGTGCACACGCTCGATGCCAGGCCAGTGTCGTTGCCTTGACACC    279
                       * ***  *    ***   *** **   **    ** *  ******  ***  ******

Glyma.09G158500.1      ACCGATTTCTGCTATGAACCTTGCAAACCCAGTGAGGATGACAAGGAAAACTACTAA       357
Glyma.09G158900.2      ACCGATTTCTGCTATGAACCTTGCAAACCCAGTGAGGATGACAAGGAAAACTACTAA       357
Glyma.14G117700.1      ACGGATTTCTGCTATGAACCTTGCAAGCCCAGTGAGGATGACAAGGAAAACTACTAA       357
Glyma.16G208900.1      AACGACTTCTGCTACAAACCTTGCAAGTCCAGAGATGACTAG---------------       327
Glyma.09G158600.1      ACCGACTTCTGCTACAAACCTTGCAAGTCCAGTGATGAAGATGATGACTAG------       330
Glyma.09G158700.1      ACCGACTTCTGCTACAAACCTTGCAAGTCCAGTGATGAAGATGATGACTAG------       330
                       *  ** ********  **********  **** ** **  *
```

Supplemental Figure S2. Heatmap showing the clustering of gene expression profiles between wild-type (Maverick) and BBi-silenced soybean seeds (BBi) at three seed developmental (E= early; M=middle; L=Late) stages.

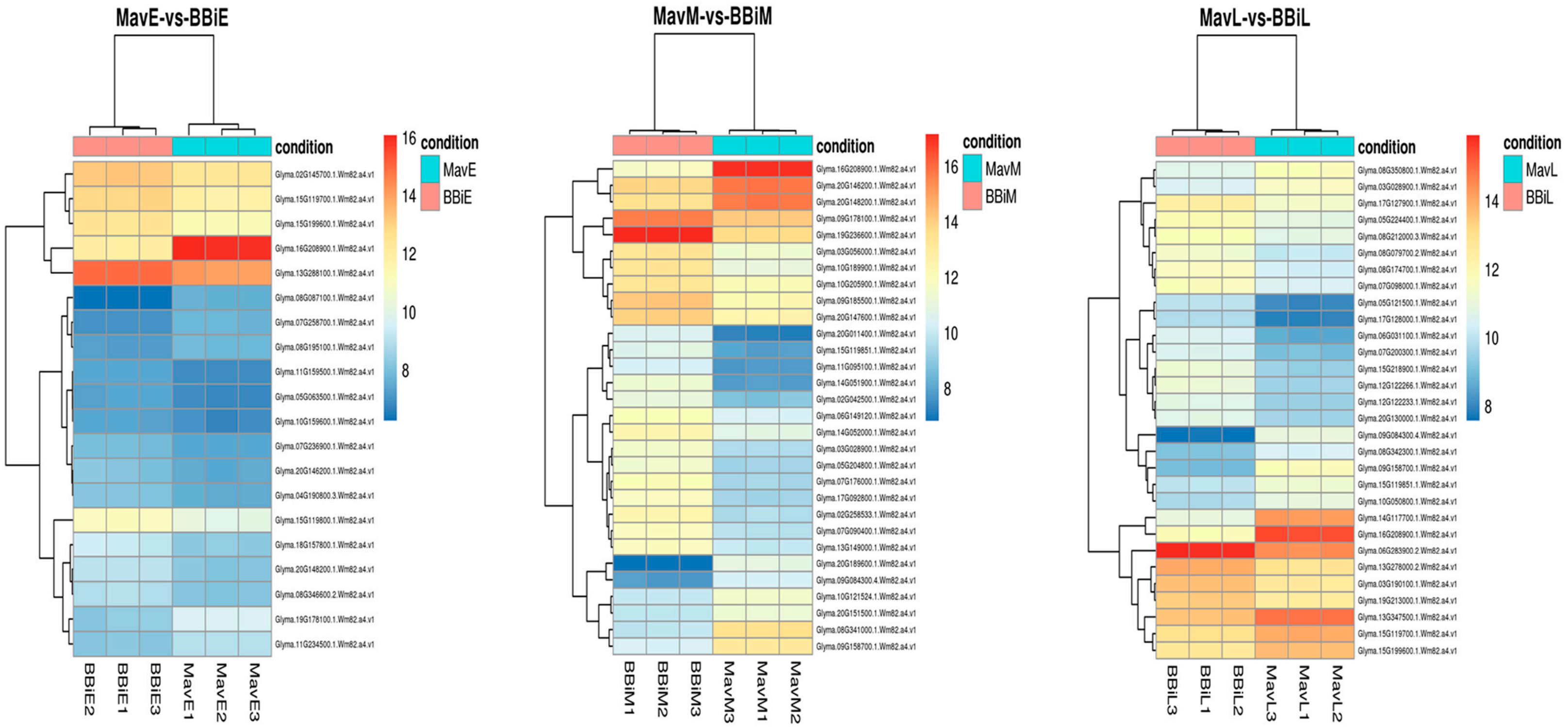

Supplemental Figure S3. Schematic diagram of the construct used for the suppression of BBi genes in soybean seeds. The RNAi cassette contains a 168 bp region of the BBi coding region cloned in an inverted repeat orientation and separated by the intron from pKannibal. The RNAi construct is under the control of the abundantly expressed soybean BBi gene. The construct also contains a gene expression cassette that includes the cauliflower mosaic virus 35S promoter, the bar-coding region and the 3' region of the nopaline synthase gene (nos).

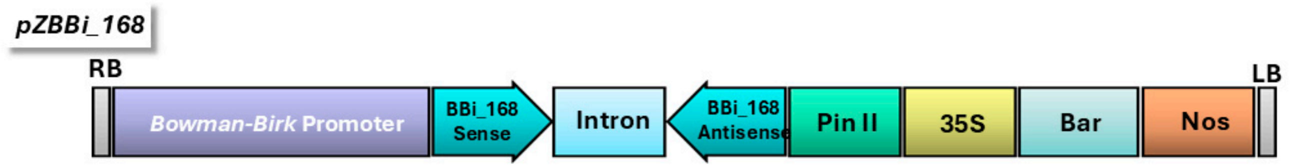

Supplemental Table S1. List of *BBi* and *KTi* genes expressed in soybean seeds. Genes exhibiting high expression levels in seeds are indicated in red font.

***BBi* genes**	***KTi* genes**
Glyma.09g158500	Glyma.01G095000
Glyma.09g158600	Glyma.08G341000
Glyma.09g158700	Glyma.08G341500
Glyma.09g158800	Glyma.08G342100
Glyma.14g117700	Glyma.08G342300
Glyma.16g208900	Glyma.16G206100
Glyma.18g231400	Glyma.16G211700
Glyma.18g231500	Glyma.16G206300

Supplemental Table S2. List of up and down regulated genes in BBi knockout line (BBi) at three seed developmental (E= early; M=middle; L=Late) stages.

	**Phytozome Auto Define**	**NCBI Definition**
**BBi_Early Stage Down Regulated**		
Glyma.16G208900	Bowman-Birk serine protease inhibitor family (Bowman-Birk_leg)	Bowman-Birk type proteinase inhibitor D-II precursor
Glyma.08G087100	SF275 - THIOREDOXIN O1, MITOCHONDRIAL-RELATED	Hypothetical protein JHK85_021278
Glyma.19G178100	Large subunit ribosomal protein L4e (RP-L4e, RPL4)	
**BBi_Early Stage Up Regulated**		
Glyma.15G119800	Hydrophobic seed protein (Hydrophob_seed)	Hydrophobic seed protein-like
Glyma.15G119700	Hydrophobic seed protein (Hydrophob_seed)	Hydrophobic seed protein precursor
Glyma.15G199600		Hypothetical protein GLYMA_15G199600v4
Glyma.20G148200	Cupin (Cupin_1) // Cupin domain (Cupin_2)	Beta-conglycinin beta subunit 2 precursor
Glyma.18G157800	Plant PEC family metallothionein (Metallothio_PEC)	Glycine max metallothionein-II protein

**BBi_Middle Stage Down Regulated**		
Glyma.12G077366		Uncharacterized protein LOC100305977 precursor
Glyma.16G208900	Bowman-Birk serine protease inhibitor family (Bowman-Birk_leg)	Bowman-Birk type proteinase inhibitor D-II precursor
Glyma.15G057600		Hypothetical protein JHK85_042156
Glyma.14G032800	Protease inhibitor/seed storage/LTP family (Tryp_alpha_amyl)	Uncharacterized protein LOC100500178 precursor
Glyma.20G189600	Geraniol 8-hydroxylase / G10H	Geraniol 8-hydroxylase
Glyma.14G048200	SF365 - PROPROTEIN CONVERTASE SUBTILISIN/KEXIN // SUBFAMILY NOT NAMED	Subtilisin-like protease
Glyma.10G021700	SF1 - CLASSICAL ARABINOGALACTAN PROTEIN 26	Classical arabinogalactan protein 26-like precursor
Glyma.13G260800		Hypothetical protein GYH30_037413
Glyma.12G097400	Plant disease resistance response protein	Dirigent protein 2
Glyma.20G044500		Hypothetical protein JHK86_055129
Glyma.11G256500	SF98 - O-METHYLTRANSFERASE // O-METHYLTRANSFERASE FAMILY PROTEIN	(RS)-norcoclaurine 6-O-methyltransferase-like
Glyma.06G050400	SF18 - T1N24.5 PROTEIN	Hypothetical protein AAZV13_06G045200
Glyma.12G135300	ATP-binding cassette, subfamily B (MDR/TAP), member 1 (ABCB1)	Putative multidrug resistance protein

Glyma.19G133600	SF8 - EXTENSIN, PROLINE-RICH PROTEIN // SUBFAMILY NOT NAMED	Gibberellin-regulated protein 35 precurso
Glyma.20G242600		Uncharacterized protein LOC100305999 precursor
Glyma.18G232500	Plant self-incompatibility protein S1 (Self-incomp_S1)	S-protein homolog 5-like
Glyma.13G110250		Uncharacterized protein LOC100306012
Glyma.08G228650	S locus-related glycoprotein 1 binding pollen coat protein (SLR1-BP) (SLR1-BP)	Hypothetical protein JHK87_022067
Glyma.04G192500	SF25 - XANTHINE-URACIL / VITAMIN C PERMEASE FAMILY MEMBER // ADENINE/GUANINE PERMEASE AZG2	Adenine/guanine permease AZG2
Glyma.08G341000	SF5 - KUNITZ FAMILY TRYPSIN AND PROTEASE INHIBITOR PROTEIN-RELATED	Kunitz-type trypsin inhibitor KTI1-like
Glyma.11G159200		Hypothetical protein JHK85_032149
Glyma.09G084300		187-kDa microtubule-associated protein AIR9 isoform X2
Glyma.10G121524	Factor independent urate hydroxylase / Uricase II	Uricase-2 isozyme 1
Glyma.20G151500	Sulfate adenylyltransferase / Sulfurylase	ATP sulfurylase 1, chloroplastic
Glyma.05G092200		Hypothetical protein JHK87_012274
Glyma.14G117700	Bowman-Birk serine protease inhibitor family (Bowman-Birk_leg)	Bowman-Birk type proteinase inhibitor precurso
Glyma.06G160300	SF3 - FAMILY NOT NAMED // DEFENSIN-LIKE PROTEIN 6	Defensin-like protein isoform 1 precursor

Glyma.15G108700	Adenylyl-sulfate reductase (thioredoxin) / Thioredoxin-dependent 5'-adenylylsulfate reductase	5'-adenylylsulfate reductase 3, chloroplastic
**BBi_Middle Stage Up Regulated**		
Glyma.06G024500	SF2 - GIBBERELLIN-REGULATED PROTEIN 1-RELATED	Gibberellin-regulated protein 1
Glyma.15G062400	Pathogenesis-related protein 1 (PR1)	Pathogenesis-related protein 1 precursor
Glyma.05G095633		Hypothetical protein GLYMA_05G095633v4
Glyma.08G235300	Trypsin and protease inhibitor (Kunitz_legume)	Trypsin inhibitor (Kunitz) family of protease inhibitors precursor
Glyma.12G200700	Wound-induced protein (DUF3774)	Uncharacterized protein LOC106795394
Glyma.14G052902	Glycine rich protein family (GRP)	Glycine-rich protein DOT1 isoform X1
Glyma.20G011500	Glycine rich protein family (GRP)	Glycine-rich protein
Glyma.15G179101		Hypothetical protein GLYMA_15G179101v4
Glyma.10G050800	SF11 - FAMILY NOT NAMED // PEROXIDASE 21	Peroxidase 21
Glyma.15G081400	SF1 - PHOSPHOLIPASE A1-IIALPHA-RELATED	Phospholipase A1-II 1
Glyma.12G122233		Hypothetical protein GLYMA_12G122233v4
Glyma.14G051900	Glycine rich protein family (GRP)	Uncharacterized protein LOC100860051 precursor

Glyma.13G301900	Wound-induced protein (DUF3774)	Uncharacterized protein LOC100796236
Glyma.09G112100	SF2 - FAMILY NOT NAMED // LATE EMBRYOGENESIS ABUNDANT PROTEIN 4-5	18 kDa seed maturation protein
Glyma.20G011400	Glycine rich protein family (GRP)	Probable H/ACA ribonucleoprotein complex subunit 1
Glyma.13G168200	SF15 - LACTOYLGLUTATHIONE LYASE GLYOXALASE I // SUBFAMILY NOT NAMED	Putative lactoylglutathione lyase
Glyma.20G011300	Glycine rich protein family (GRP)	Major prion protein
Glyma.06G149120	Alginate lyase (Alginate_lyase2)	24 kDa seed coat protein precursor
Glyma.15G119851	Hydrophobic seed protein (Hydrophob_seed)	Uncharacterized protein LOC102667340
Glyma.02G042500	SF34 - CHITINASE-RELATED // BASIC ENDOCHITINASE B	Chitinase class I precursor
Glyma.13G149000	SF11 - LATE EMBRYOGENESIS ABUNDANT PROTEIN (ATECP63)-RELATED	Maturation polypeptide
Glyma.14G052000	Glycine rich protein family (GRP)	Uncharacterized protein LOC100804226 precursor
Glyma.20G147600	SF7 - LEA PROTEIN-RELATED	Seed maturation protein PM25
Glyma.11G095100	Glucan endo-1,3-beta-D-glucosidase / Laminarinase	Glucan endo-1,3-beta-glucosidase precursor
Glyma.10G205900	SF5 - AWPM-19-LIKE FAMILY PROTEIN	Maturation protein PM3
**BBi_Late Stage Down Regulated**		

Glyma.01G123700		Hypothetical protein GLYMA_01G123700v4
Glyma.02G051700	SF79 - BETA-GALACTOSIDASE RELATED // BETA-GALACTOSIDASE 3	Beta-galactosidase 3
Glyma.11G049600	SF3 - FAMILY NOT NAMED // PEROXIDASE 35-RELATED	Peroxidase 51
Glyma.17G203450	SF3 - MOLYBDATE TRANSPORTER 1	Hypothetical protein GLYMA_17G203450v4
Glyma.15G048500	SF409 - MITOGEN-ACTIVATED KINASE KINASE KINASE	Hypothetical protein GLYMA_15G048500v4
Glyma.07G069500	SF9 - FAMILY NOT NAMED // CYTOCHROME P450 86A2-RELATED	Cytochrome P450 86A8
Glyma.16G208900	Bowman-Birk serine protease inhibitor family (Bowman-Birk_leg)	Bowman-Birk type proteinase inhibitor D-II precursor
Glyma.09G084300		187-kDa microtubule-associated protein AIR9 isoform X2
Glyma.09G158800	Bowman-Birk serine protease inhibitor family (Bowman-Birk_leg)	Bowman-Birk type proteinase inhibitor C-II
Glyma.20G044500		Hypothetical protein JHK86_055129
Glyma.14G117700	Bowman-Birk serine protease inhibitor family (Bowman-Birk_leg)	Bowman-Birk type proteinase inhibitor precurso
Glyma.13G104100	SF300 - AMINE OXIDASE	Probable polyamine oxidase 5
Glyma.15G119700	Hydrophobic seed protein (Hydrophob_seed)	Hydrophobic seed protein precursor
Glyma.15G119851	Hydrophobic seed protein (Hydrophob_seed)	Uncharacterized protein LOC102667340

Glyma.08G350800	Beta-amyrin 24-hydroxylase (CYP93E1)	Beta-amyrin 24-hydroxylase
Glyma.03G028900	SF35 - FAMILY NOT NAMED // PECTINESTERASE/PECTINESTERASE INHIBITOR 36-RELATED	Probable pectinesterase/pectinesterase inhibitor 36
**BBi_Late Stage Up Regulated**		
Glyma.18G009900	Mitotic spindle assembly checkpoint protein MAD2 (MAD2)	Mitotic spindle checkpoint protein MAD2
Glyma.14G080701	C-TERMINALLY ENCODED PEPTIDE 1	Precursor of CEP5-like
Glyma.16G170100	Extensin-like region (Extensin_2)	Extensin-like protein precurso
Glyma.02G060900	Domain of unknown function (DUF4228) (DUF4228)	Uncharacterized protein LOC100809248
Glyma.15G097800	Protein arginine N-methyltransferase 6 [EC:2.1.1.-] (PRMT6)	Hypothetical protein GLYMA_15G097800v4
Glyma.05G182800	Abieta-7,13-dien-18-ol hydroxylase / CYP720B1	Cytochrome P450 CYP736A12
Glyma.06G031100		Uncharacterized protein LOC100306259
Glyma.15G218900	Pathogenesis-related protein Bet v I family (Bet_v_1)	Uncharacterized protein LOC100527208
Glyma.12G122266		Uncharacterized protein DDB_G0286299-like isoform X2
Glyma.05G121500	SF6 - VACUOLAR IRON TRANSPORTER HOMOLOG 2.1	Nodulin-21
Glyma.08G174700	SF195 - GLUTATHIONE S-TRANSFERASE, GST, SUPERFAMILY, GST DOMAIN CONTAINING	Glutathione S-transferase GST 9

Glyma.17G128000	Malate synthase (E.D. 2.3.3.9, aceB, glcB)	Malate synthase, glyoxysomal
Glyma.06G283900	SF49 - LATE EMBRYOGENESIS ABUNDANT PLANTS LEA-RELATED // COLD-REGULATED PROTEIN 15B	Seed biotin-containing protein SBP65
Glyma.03G190100	SF7 - PROGRAMMED CELL DEATH 4	Uncharacterized protein LOC100785774
Glyma.07G200300	SF158 - 17.6 KDA CLASS I HEAT SHOCK PROTEIN 1-RELATED	Putative class I heat shock protein
Glyma.13G278000	SF4 - FAMILY NOT NAMED // DEFENSIN-LIKE PROTEIN 13-RELATED	Defensin-like protein
Glyma.05G224400	Zinc finger C-x8-C-x5-C-x3-H type (and similar) (zf-CCCH)	C3H-type zinc finger protein
